# Study of the dual biological impacts of aqueous extracts of normal and gamma-irradiated *Galleria mellonella* larvae

**DOI:** 10.1016/j.jtumed.2021.12.016

**Published:** 2022-03-29

**Authors:** Rehab Sayed, Nessren A. Safwat, Basma H. Amin, Mohammed Yosri

**Affiliations:** a Natural Product Department, National Center for Radiation Research and Technology, Egyptian Atomic Energy Authority, Cairo, Egypt; b The Regional Center for Mycology and Biotechnology, Al-Azhar University, Cairo, Egypt

**Keywords:** دودة الشمع, أشعة جاما, النشاط الأنزيمي, النشاط المضاد للميكروبات, تأثير مضاد للورم, Antimicrobial activity, Antitumor effect, Enzymatic activity, *Galleria mellonella*, Gamma-radiation

## Abstract

**Objectives:**

*Galleria mellonella* assimilates beeswax using many gut enzymes; however, high doses of gamma radiation have been used to eradicate such pests, affecting its life cycle. *In vitro* studies of irradiated extracts of *G. mellonella* against bacterial species as well as three tumour cell lines are demonstrated in the present study. The antibacterial and antitumour effects are compared with those of the non-irradiated *Galleria mellonella* larval extract.

**Methods:**

The effect of different dose levels of gamma irradiation, ranging from 2 to 8 Gy, was tested on *G. mellonella* lipase, protease, and acid phosphate activities. The antimicrobial activity of un-irradiated and irradiated *G. mellonella* larval extracts was tested against different gram-positive and gram-negative bacteria and some fungi. The antitumour action was tested against different tumour cell lines. A cytotoxicity assay was performed on normal and irradiated larval extracts against normal human lung fibroblast cells. A microscopic examination of *Streptococcus* mutants and HepG-2 was performed using transmission and scanning electron microscopy.

**Results:**

Optimum results were obtained at 6 Gy, which enhanced maximum enzymatic activity. Maximum antimicrobial activity was obtained against *Streptococcus* mutants with MIC 31.25 μg/ml at a dose of 6 Gy. A microscopic examination depicted an apoptotic process for irradiated *G. mellonella* larvae with either *Streptococcus* mutants or HepG-2.

**Conclusion:**

The present study shows a synergistic relationship between the *G. mellonella* larval extract and a 6 Gy radiation dose for further biomedical applications.

## الملخص

**أهداف البحث:**

تهضم الغاليريا الميلونيلا (دودة الشمع) شمع العسل باستخدام العديد من إنزيمات الأمعاء، ولكن بجرعات عالية من أشعة جاما يتم القضاء على مثل هذه الآفات التي تؤثر على دورة حياتها. الدراسات المخبرية للإشعاع المستخلص من دودة الشمع ضد الأنواع البكتيرية بالإضافة إلى ثلاثة أنواع من الخلايا السرطانية تمت تجربته في هذه الدراسة. تمت مقارنة التأثيرات المضادة للبكتيريا والسرطان مع مستخلص اليرقات غير المشع من دودة الشمع.

**طرق البحث:**

تم اختبار تأثير مستويات الجرعات المختلفة من أشعة جاما التي تتراوح من 2 إلى 8 جيجا بايت على أنزيمات دودة الشمع الليباز، البروتياز، وأنشطة الفوسفات الحمضي. تم إجراء النشاط المضاد للميكروبات لمستخلصات يرقات دودة الشمع غير المشعة والمعرضة للإشعاع ضد أنواع مختلفة من البكتيريا سالبة الجرام وإيجابية الجرام وبعض الفطريات. تم عمل مضاد للورم ضد مختلف أنواع الخلايا السرطانية المستخدمة. تم إجراء فحص السمية الخلوية لمستخلصات اليرقات الطبيعية والمعرضة للإشعاع الخلايا الليفية الرئوية البشرية الطبيعية. الفحص المجهري باستخدام الإرسال والمسح تم إجراء الفحص المجهري الإلكتروني للطفرات العقدية والخلايا السرطانية هب-جي2.

**النتائج:**

تم الحصول على أفضل النتائج عند 6 جيجا بايت مما عزز النشاط الأنزيمي الأقصى. تم الحصول على أقصى نشاط مضاد للميكروبات ضد طفرات المكورات العقدية باستخدام التركيز الأدنى المثبط وهو 31,25 مايكروغرام/مل بجرعة 6 جيجا بايت. أظهر الفحص المجهري عملية موت الخلايا المبرمج ليرقات دودة الشمع المعرضة للإشعاع مع أي من طفرات المكورات العقدية أوالخلايا السرطانية هب-جي2.

**الاستنتاجات:**

أظهر التقرير الحالي علاقة تآزرية بين مستخلص يرقات دودة الشمع والجرعة الإشعاعية 6 جيجا بايت لمزيد من التطبيقات الطبية الحيوية.

## Introduction

Mouse models are the most common tools to test biological effects, including antimicrobial and antitumour effects, using standard protocols that ensure animal welfare in research. Therefore, efficient alternative tools have been developed for different experiments, including those on *Caenorhabditis elegans, Artemia salina, Drosophila melanogaster, and Galleria mellonella* (*G. mellonella*), to obtain reliable results.^1^, ^2^, ^3^

Many laboratories have developed methods to screen whole animal extractions to find promising and suitable inflammatory, anticancer, antiviral, as well as antimicrobial effects that develop resistance to known antibiotics.^4^ *G. mellonella* has recently been used to test synergistic mechanisms with anti-inflammatory drugs such as dexamethasone and licofelone.^5^^,^^6^

Insects feed on different compact sources and need degrading enzymes, such as proteases, lipases, and acid phosphatases, to assimilate these components into a digestible form. Termites’ guts contain microorganisms that produce a group of enzymes to digest wood into simple monomers; these enzymes have been used in recent applications and industries.^7^^,^^8^ Many insects, such as *Tenebrio molitor* and *Ulomoides dermestoides* beetles, are used as cuisine in many European and Latin American countries due to their nutritional value in the form of proteins and minerals.^9^ *Odontotermes formosanus* has recently been used to find innovative biomedical applications related to certain bacterial resistance to regular drugs.^10^ The larvae of *Lucilia sericata* have been applied in maggot treatment due to the formation of antimicrobial factors in the larvae body, which are secreted on the outer surface; these factors permeate the cell membrane of the microbe, leading to electrolyte depolarisation.^11^ Various extraction methods for insects using many solvents, such as methanol and hexane, have been used to maintain maximal activity of enzymatic products due to their bioactivities, including antioxidant, antidiabetic, and antihyperlipedimia.^12^ *G. mellonella* assimilates beeswax using many gut enzymes. Furthermore, the high doses of gamma radiation that are used to eradicate such pests affect their life cycles.^13^

Chitosan oligosaccharides present in insects’ exoskeletons. They have several biological activities, including anticancer, anti-obesity, and anti-hypertension, and modulate mitogen-activated protein kinases and the AMP-activated protein kinase pathways.^14^ Royal jelly is a bee product with many medical uses, including antimicrobial, antioxidant, and antitumour effects. It contains various bioactive compounds, including 10-hydroxydecanoic acid and 24-methylenecholesterol.^15^ A group of researchers have suggested a combination of extracts from *P. americana* with traditional Chinese medicine for medical applications, including antitumour activity.^16^ Certain products have dual antitumour agents and a natural insecticide, such as fraxinellone, which is a degraded limonoid derived from the root bark of the Dictamnus plant. Fraxinellone has hepatoprotective and antitumour action by disrupting the insect cytokine growth-blocking peptide in the EGF family.^17^ Radiation therapy is one of three essential tools for the treatment of tumours. It has an anticancer effect in cancer therapy in combination with other known compounds that are considered to be alternative effective tools.^18^ A combination of gamma radiation with entemo-pathogenic fungi to combat *G. mellonella* larvae has shown promising results at higher doses reaching 150 Gy.^19^ The aim of the current work is an *in vitro* testing of an irradiated larval *G. mellonella* extract against bacterial species and three tumour cell lines, which are investigated for the first time in the present study. The antibacterial and antitumour effects are compared with those of a non-irradiated *G. mellonella* larvae extract.

## Materials and Methods

### *G. mellonella* rearing

Wax moth culture was grown on an artificial medium that comprised 22% corn groats, 22% wheat-flour, 11% milk powder, 11% honey, 11% glycerol, 5.5% yeast powder, and 17.5% bee wax. Rearing was done by placing the eggs in a transparent glass-rearing jar containing 250 gm of the previously prepared medium, closed tightly with a double muslin layer to prevent the escape of neonatal larvae, and incubated under certain rearing conditions (28° ± 2C and 65 ± 5% relative humidity) with a photoperiod (L: D) 8:16. Food was added once a week until the larvae grew to the 6th instar; they were then collected for further experiments.^20^

### Irradiation technique

Healthy (creamy coloured 6th instar) larvae were collected and irradiated with 2, 4, 6, and 8 Gy using a γ Cell Unit (Cs^137^ source) with a dose rate of 0.645 rad/s and placed at the National Center for Radiation Research and Technology, Egyptian Atomic Energy Authority.

### Larvae extract preparation

One gram of the irradiated and normal (un-irradiated) larvae were surface-sterilised and incubated in 200 μl dH_2_O in sterile containers for 1 h at 30 °C in darkness. We then filled up the volume for 1 ml/1 g larvae. The larvae were cut and squeezed in dH_2_O, incubated in it, and centrifuged at 10,000×*g* for 5 min to eliminate debris, leaving the supernatant for testing.^21^

### Biochemical analysis

Total proteins were estimated by the Bradford method,^22^ in which a protein reagent was prepared by dissolving 100 mg of Coomassie Brilliant Blue in 50 ml 95% ethanol; 100 ml 85% (W/V) phosphoric acid were then added, and the resulting solution diluted to a final volume of 1 litre. For the preparation of a standard curve, 50 μl of serial concentrations containing 10 to 100 μg bovine serum albumin were pipetted into test tubes (for the estimation of the larval extracts, 50 μl were used instead of the bovine serum albumin); the volume in the test tube was adjusted to 1 ml with a phosphate buffer (0.1M, pH 6.6). Then, 5 ml of the protein reagent were added to each test tube, and the contents mixed well. The absorbance was measured at 595 nm after 2 min and before 1 h against a blank (1 ml of phosphate buffer and 5 ml protein reagent).^22^

Protease activity was determined according to **Zhang et al.**,^23^ with some modifications, by measuring the elevation in the free amino-acids split from the substrate protein (albumin) during 1-hone-hour incubation at 30 °C; the amino acids were assayed using ninhydrin; the reaction mixture comprised 100 μl larvae homogenates, 1 ml of 0.1 M phosphate buffer (pH 8), and 100 μl of 0.5% bovine serum albumin. The reaction was stopped by adding 1.2 ml 20% tri-chloro-acetic acid (TCA). Fifteen minutes after the reaction was stopped, the mixture was centrifuged at 3000 r.p.m. for 20 min, and the supernatant used to measure the quantity of the amino acids produced. The amino acids were calorimetrically assayed using ninhydrin reagent; 100 μl of the previous supernatant was added to 1.9 ml of ninhydrin-citrate (pH 5.5), 0.2 ml of 0.5 M citrate buffer (pH 5.5), and 1.2 ml glycerol. The mixture was heated in a boiling water bath for 12 min and cooled by tap water. The developed colour was read at 570 nm against a reagent blank (which contained everything and 100 μl dH_2_O water instead of the supernatant). D, L alanine was used as the standard, and the amino acids were expressed as μg alanine/min/g.b.wt.^24^

Lipase activity was evaluated following Choi et al.,^25^ with a slight modification that was based on a determination of the decline in the ester content of triolein as the substrate. The lipid emulsion was prepared by blending 4 g triolein, 7.74 g triton–x 100, 0.22 g CaCl, and 0.234 g sodium chloride, and then filled to 100 ml with 0.2 mol/L tris-buffer (pH 7.5). One millilitre of substrate emulsion, 100 μl larvae extract, and 0.4 ml 0.2 M tris buffer (pH 7.5) were incubated at 35 °C for exactly 5 min. At the end of the incubation period, 4 ml of a mixture consisting of 4 ml iso-propanol and 2 ml 1M H_2_SO_4_ were added to stop the enzyme reaction. The reaction products were extracted in 5 ml n-heptane after vigorous stirring, and the mixture allowed to stand for 5 min. One millilitre of the n-heptane layer was transferred to a test tube and the optical density of the ester content determined relative to that of the standard.^25^

Acid phosphatase was determined following Tietz.^26^ In this method, the phenol released by the enzymatic hydrolysis of disodium phenylphosphate reacts with 4-aminoantipyrine; the addition of potassium ferricyanide produces a characteristic brown colour. The reaction mixture comprised 1 ml citric buffer (pH 4.9), 1 ml of 0.01M disodium phenylphosphate (substrate), and 0.1 ml larvae extract mixed and incubated for exactly 30 min at 37 °C. At the end of the incubation period, 0.8 ml of 0.5N Na0H was added to stop the reaction. Then, 1.2 ml of 0.5N NaHCO_3_ was added, followed by the addition of 1 ml of 4-aminoantipyrine solution (1%) and 1 ml potassium ferricyanide (0.5%). The produced colour was measured immediately at 510 nm. Enzyme activity is expressed in units (U): 1 unit will hydrolyse 1.0 μmol of p-nitophenyl phosphate/min at 37 °C and pH 4.8.^26^

### Antimicrobial activity and minimum inhibitory concentration (MIC) for the extract

To test the antimicrobial action of the aqueous extracts of normal and irradiated *G. mellonella* larvae against test organisms, the Agar diffusion method was used, with 100 μl of extract filtrate to fill the holes. At the end of the incubation period, the inhibition zones were measured; the sets were compared with the standard drug. Serial dilutions of the effective dose were prepared and tested for various test organisms.^27^

### Cell culture and antitumour assay

The MCF-7 (ATCC® HTB-22™), PC3 (ATCC® CRL-1435 ™), and HepG-2 (ATCC® HB-8065™) cell lines were kindly offered by, and obtained from The Regional Center for Mycology and Biotechnology – Al Azhar University. The cells were grown in normal culture conditions in Dulbecco's modified Eagle's medium, supplemented with 10% heat-inactivated foetal bovine serum, 1% L-glutamine, HEPES buffer, and 50 μg/ml gentamycin. The cells were preserved at 37 °C in a humidified atmosphere with 5% CO_2_ for further investigations.

Regular and treated cells were incubated with DMSO; the media were then aspirated and stained with crystal violet. The stain was removed, glacial acetic acid (30%) was added to all wells, and the absorbance of the plates was then measured at 490 nm.^28^

### Cytotoxicity assay

To test the safety of the larval extracts, normal human lung fibroblast (WI38) cell lines were used. The cells were floated in 100 μl/well, 96-well tissue culture plates at a concentration of 6 × 10^3^ cell/well, then incubated for 24 h. Then, 10 μl of extracts at concentrations of 1000, 500, 250, 125, 62.5, 31,25, 15.6, 7.8, 3.9, 2, and 0 μg/ml, diluted in 0.5% DMSO, were added to the cells in the plates. The plates were incubated for 24 h in a CO_2_ incubator, where the cells were incubated with and without larval extracts. After incubation, crystal violet was added and then washed with distilled water; 30% glacial acetic acid was then added to detect viable cells at 490 nm.^28^

### Cell imaging using inverted microscopy

After 3 days of incubation, the plates were examined using an inverted microscope, and the images captured using a digital CCD camera (Zeiss, Berlin, Germany) in the Regional Center for Mycology and Biotechnology.

### Transmission electron microscopy

*Streptococcus mutants* and HepG-2 cells were treated with irradiated larval extracts and then fixed with 2.5% glutaraldehyde for 2 h. The samples were then processed with 2% osmium tetroxide for 2 h, and the blocks stained in 1% uranyl acetate and dehydrated with a graded ethanol series. The samples were then embedded with resin. The samples were sectioned using an ultra-microtome (Leica, Wetzkar, Germany), and the sections then viewed on a transmission electron microscope (JOEL, Tokyo, Japan).^29^

### Scanning electron microscopy

Scanning electron microscopy was used to investigate the outer surface of normal and treated HepG-2 cells. Fixed samples were dehydrated in an ethyl alcohol series, coated with gold, and then examined using a scanning microscope (JOEL, Tokyo, Japan) in the Regional Center for Mycology and Biotechnology.^30^

### Statistical analysis

Data were represented as means ± standard deviations of means (SD) and analysed using GraphPad Prism (version 5.0, San Francisco, USA). *p* = 0.05 was regarded as statistically significant (an unpaired two-sided Student’s t-test and, one-way analysis (ANOVA) were used).

## Results

### Enzymatic assay for normal and irradiated *G. mellonella* larval extracts

**Figure 1:**
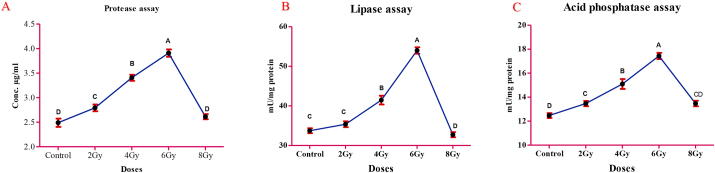
Effect of gamma radiation doses on some enzymatic activities of the *G. mellonella* larval extract. A: protease activity; B: lipase activity; and C: acid phosphatase activity. Values represent the mean ± SEM of 3 replicates for each treatment. Maximum value was reported at dose 6 Gy in all tested enzymes. Different letters mean statistical significance at P < 0.05 (Tukey pairwise comparisons test).

Variations in the concentrations of extracellular protease, lipase, and acid phosphatase of the *G. mellonella* extract resulted from irradiation by 2, 4, 6, and 8 Gy, as depicted in Figure 1A, B, and C. A dramatic elevation in the concentration of enzymes was observed relative to the control sample on increasing the dose to 6 Gy. Moreover, increasing the dose level above 6 Gy led to a significant decline in the concentrations of protease, lipase, and acid phosphatase (*p* < 0.05).

### Antimicrobial activities and MIC of normal and irradiated *G. mellonella* larval extracts

The mean inhibition versus *Streptococcus mutants* was higher compared to other test microorganisms, whereas at a dose of 6 Gy, maximal inhibition was reported against *Bacillis subtilis*, *A fumigatus*, *Escherichia coli, Candida albicans*, and *A. fumigatus*, compared to the other doses. Furthermore, the highest inhibitory activity observed was 18.5 ± 0.47 mm against *Streptococcus mutants*, while the lowest action was 14.7 ± 2.3 mm against *Aspergillus fumigatus*; there was no effect on *Pseudomonas aeruginosa* at all doses, as shown in Table 1.

**Table 1:** Mean zone of inhibition in mm ± standard deviation of samples against some microorganisms using 1 mg/ml concentration on using different gamma radiation doses.

Sample Tested microorganisms	C	0	2	4	6	St.
*FUNGI*						Amphotericin B
*Aspergillus fumigatus* (RCMB002008)	9.30 ± 2.30	11.2 ± 2.30	12.6 ± 2.30	12.7 ± 2.30	**14.7 ± 2.30**	23.7 ± 0.82
*Candida albicans* (RCMB 005003)	11.4 ± 1.70	11.9 ± 0.81	13.4 ± 0.69	19.3 ± 2.20	**16.9 ± 1.80**	25.4 ± 1.30
Gram positive Bacteria:						*Ciprofloxacine*
***Streptococcus mutants* (ATCC35668)**	**14.8 ± 0.96**	**16.1 ± 1.40**	**17.9 ± 2.40**	**15.9 ± 0.96**	**18.5 ± 0.47**	23.8 ± 0.58
*Bacillis subtilis* (ATCC6633)	13.7 ± 2.10	14.7 ± 0.36	14.4 ± 0.84	14.9 ± 1.00	**17.2 ± 0.76**	32.4 ± 1.70
Gram negative Bacteria:						*Ciprofloxacine*
*Pseudomonas aeruginosa* (ATCC27853)	NA	NA	NA	NA	NA	17.3 ± 2.30
*Escherichia coli* (ATCC25922)	12.9 ± 1.90	14.2 ± 2.20	14.7 ± 0.74	11.9 ± 2.10	**16.2 ± 1.60**	19.9 ± 1.90

∗N. A: No activity; ATCC: American type culture collection; RCMB: Regional Center of Mycology and Biotechnology.

The MICs of the sample at a dose of 6 Gy were selected for further testing, as shown in Table 2. Strep*tococcus mutants* showed the lowest MIC of 31.25 μg/ml, followed by *Candida albicans and Escherichia coli*, with a MIC of 62.5 μg/ml. Furthermore, *Aspergillus fumigatus* showed the highest MIC of 250 μg/ml.

**Table 2:** Antimicrobial activity as MICS (μg/ml) of tested samples against tested microorganisms at a dose of 6 Gy of gamma radiation.

Sample Tested microorganisms	6	St.
*FUNGI*		Amphotericin B
*Aspergillus fumigatus* (RCMB002008)	250	1.95
*Candida albicans* (RCMB 005003)	62.5	0.98
Gram Positive Bacteria:		*Ciprofloxacine*
***Streptococcus mutants* (ATCC35668)**	**31.25**	0.49
*Bacillis subtilis* (ATCC6633)	125	1.95
Gram negative Bacteria:		*Ciprofloxacine*
*Pseudomonas aeruginosa* (ATCC27853)	NA	0.98
*Escherichia coli* (ATCC25922)	62.5	0.49

∗N. A: No activity; ATCC: American type culture collection; RCMB: Regional Center of Mycology and Biotechnology.

### Antitumour activities of normal and irradiated *G. mellonella* larval extracts

**Figure 2:**
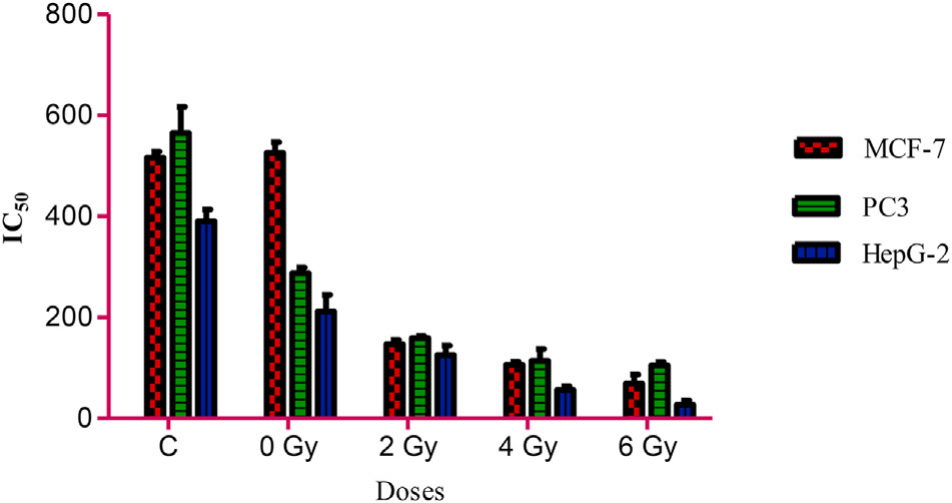
Anticancer activity of *G. mellonella* larval extract against tumour cells (MCF-7, PC-3, and HepG-2). Sample on irradiation with 2, 4, and 6 Gy, compared with normal cells (control) and extract alone (0 dose), showing the role of radiation in enhancing the antitumour activity of the *G. mellonella* larval extract; the concentrations were tested at 500, 250, 125, 62.5, 31.25, and 15.6 μg/ml.

The highest antitumour activities were reported with the sample irradiated at 6 Gy, with IC_50_ values of 80.6 ± 1.5, 110.1 ± 1.2, and 30.4 μg/ml against MCF-7, PC-3, and HepG-2 cells, respectively, followed by 80.9 ± 1.9, 100.3 ± 1.9, and 50 ± 0.4 μg/ml against MCF-7, PC-3, and HepG-2 cells, respectively, with the extract irradiated at 4 Gy. However, the *G. mellonella* extract alone had minimal antitumour activity against the tested cell lines. Collectively, the highest activity against the HepG-2 cells was obtained on irradiation at 6 Gy (P < 0.05), as shown in Figure 2.

### Cytotoxicity activities of normal and irradiated *G. mellonella* larval extracts

To test the safety of the normal and irradiated samples against normal human lung fibroblast cells, where the CC_50_ for the normal extract was >1000 μg/ml, the CC_50_ values were >1000 μg/ml, 246.79 ± 8.07 μg/ml, 400.26 ± 13.48 μg/ml, and 290.93 ± 9.21 μg/ml for the extracts irradiated with doses of 2 Gy, 4 Gy, 6 Gy, and 8 Gy, respectively, which indicated safety for the normal and irradiated larval extracts.

### Examination of variation in the morphology of HepG-2 on using normal and irradiated *G. mellonella* larval extracts

**Figure 3:**
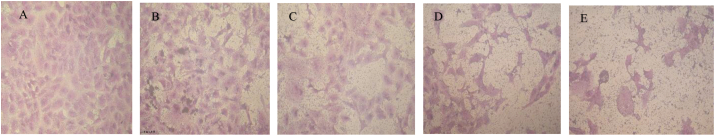
Morphological observations of the *G. mellonella* larval extract against HepG-2 cells after treatment with different doses of radiation. (A) control cells (0.1% DMSO); (B) cells treated with extract alone; (C) cells treated with sample irradiated with 2 Gy; (D) cells treated with sample irradiated with 4 Gy; and (E) Cells treated with extract irradiated with 6 Gy; inverted microscope used at 100 X.

HepG-2 cells were examined under an inverted microscope for morphological variation. Normal HepG-2 cells were flattened, polygonal, and aligned in a monolayer sheet. In the HepG-2 cells treated with the *G. mellonella* larval extract, a slight scattering of cells was observed, while treatment with a sample irradiated at 2 Gy led to deformation of some cells and disruption of the monolayer sheet. Finally, at a dose of 6 Gy, complete apoptosis as well as a decrease in the number of cells were observed, as presented in Figure 3.

### Ultra-structure examination of HepG-2 after treatment with normal and irradiated *G. mellonella* larval extracts

**Figure 4:**
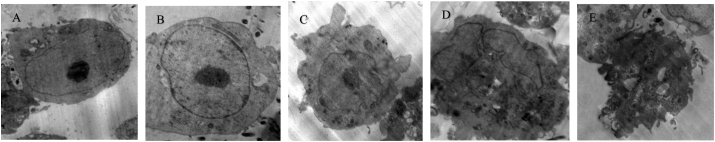
Morphological observations of *G. mellonella* extract against HepG-2 cells after treatment with different doses of radiation. (A) control cells (0.1% DMSO); (B) tumour cells treated with extract alone, with enlarged structure; (C) tumour cells treated with sample irradiated with 2 Gy, with irregular outer surface; (D) cells treated with sample irradiated with 4 Gy start apoptosis; and € cells treated with sample irradiated with 6 Gy, showing complete distortion of internal organelles; transmission electron microscope used (magnification = 5000×).

Tumour cells were investigated without treatment, and showed regular structures; this was followed by treatment with the extract alone, which led to a slight enlargement of the cells. However, treatment using 2 Gy led to irregular cell and cell membrane morphology. Moreover, a dose of 4 Gy led to the destruction of internal organelles and started an apoptosis process. Last, at a dose 6 Gy, complete distortion of cells could be observed using transmission electron microscopy, as shown in Figure 4.

### Ultra-structure examination of *Streptococcus mutants* after treatment with normal and irradiated *G. mellonella* larval extracts

**Figure 5:**
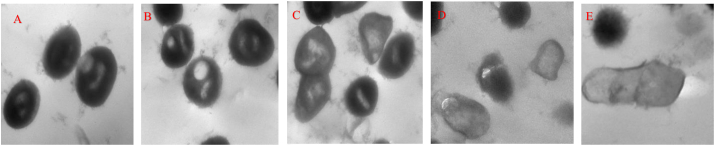
Morphological examinations of *Streptococcus mutans*. (A) *S. mutans* control cells; (B) large vacuoles could be observed after treatment with extract alone; (C) slight defects of *S. mutans* structures observed after treatment with sample irradiated with 2 Gy; (D) *S. mutans* treated with sample irradiated with 4 Gy, with deformation of shape and significant lysis of organelles; and (E) *S. mutans* treated with sample irradiated with 6 Gy, showing complete lysis of cell wall and loss of cytoplasmic organelles; transmission electron microscope used (magnification = 80,000×).

*Streptococcus mutants* were examined without treatment, and showed a regular structure; this was followed by treatment with the extract alone, which enhanced the formation of large vacuoles inside the bacterial cells. Furthermore, irregular cell structure could be observed after treatment using 2 Gy. Moreover, a dose of 4 Gy led to a noticeable destruction of cell organelles. Finally, at a dose 6 Gy, complete lysis of bacterial cells could be observed using transmission electron microscopy, as shown in Figure 5.

### Surface examination of HepG-2 on using normal and irradiated *G. mellonella* larval extracts

**Figure 6:**
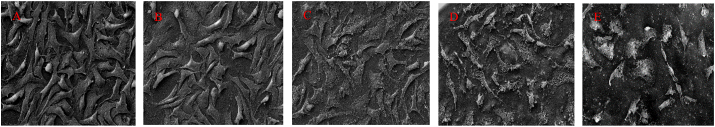
Surface examinations of cultured HepG-2 cells. (A) control (0.1% DMSO), aggregated cells with regular borders; (B) a dissociation of cells could be observed after treatment with extract alone; (C) slight shrinkage of cells observed after treatment with sample irradiated with 2 Gy; (D) cells treated with sample irradiated with 4 Gy, with rough irregular borders and shape; and (E) cells treated with sample irradiated with 6 Gy, showing complete distortion of cells; scanning electron microscope used (magnification = 5000×).

The cells were examined without treatment and showed an ordinary shape; this was followed by treatment with the extract alone, which promoted the disjunction of the cells. However, treatment using 2 Gy led to irregular surfaces and a slight contraction of the cells. Furthermore, a dose of 4 Gy caused more cell shrinkage and irregular shapes and surfaces. Last, at a dose of 6 Gy, complete cell damage could be observed using scanning electron microscopy, as shown in Figure 6.

## Discussion

Gamma radiation with a dose range of 8–75Gy has been used alone or with surgery, with many restrictions, in treating many types of breast, colon, and prostate cancers.^31^ Moreover, some trials have reported that doses above 18 Gy may lead to tumour rejection, with certain limitations.^32^ Recently, the use of natural extracts has been reported to have promising antimicrobial action.^33^ Microbial infections and tumours are considered to be major causes of death globally. There is an increasing need for an effective therapy to combat such threats; one successful tool is combinational therapy.^34^ In the current study, minimal doses of radiation were applied to *G. mellonella* larvae, which were extracted using water for further testing. The haemolymph of *G. mellonella* has been reported to have antimicrobial action against different fungi, gram-positive, and gram-negative bacteria, including *Legionella gormanii*.^35^ In the present results, the *G. mellonella* larval extract has maximal efficacy against *Streptococcus mutants* at a dose of 6 Gy of gamma radiation. Several mechanisms have been described to enhance antimicrobial activity on using different doses of blue light, resulting in cell membrane as well as DNA damage in bacteria due to oxidative enzymes.^36^ In the present study, the protease, lipase, and acid phosphatase enzymes produced by the *G. mellonella* larvae reached the maximal level at 6 Gy.^37^ Wenlong et al. reported that 200 Gy irradiation disrupted the enzyme levels of *P. xylostella*, leading to body damage, while long-wave ultraviolet light was reported to increase oxidative enzymes in *Dendrolimus tabulaeformis*.^38^ In the current study, 2, 4, 6, and 8 Gy of gamma radiation doses were applied to *G. mellonella* larvae, followed by aqueous extraction and testing on prostate cancer (PC-3), breast cancer (MCF-7), and liver cancer (HepG-2) cell lines. Promising results were obtained, especially with the HepG-2 cells at 6 Gy, with minimal cytotoxicity against normal human lung fibroblast (WI38) cells, in accordance with other research groups^31^^,^^39^ who investigated the use of low radiation doses to enhance the antitumour immune response against colorectal cancer liver. Moreover, Aneta et al.^40^ reported that *G. mellonella* had hydrolysis enzymes that induced an apoptosis process in *Candida albicans.* Ying et al.^41^ reported that the glutathione S-transferase enzyme increased apoptosis markers in irradiated hepatocellular carcinoma cells, consistent with the current results from the inverted microscope that show apoptosis of HepG-2 cells treated with a sample irradiated at 6 Gy. Moreover, Maxwell et al.,^42^ who used *Xenorhabdus nematophilus* infected with a *G. mellonella* water extract, reported success against soil bacteria, which is consistent with the current results that confirmed the activity of *G. mellonella* against *Candida albicans, Aspergillus fumigatus, Bacillus subtilis,* and *Escherichia coli*, as well as the remarkable action on *Streptococcus mutants* on irradiation with 6 Gy. Microscopic imaging was used as an effective and global testing method for the diagnosis of tumours.^43^ In the present study, both transmission and scanning electron microscopes were used to investigate the antibacterial and antitumour action of a *G. mellonella* water extract, on irradiation with 6 Gy, of destroying cell structures and inducing an apoptosis process. However, other studies have focused on using plant extracts in medical applications as well as developing effective tools against cancer and microbes.^44^^,^^45^ Thus, this has satisfied the need for more innovative methods to be applied using other alternative therapies.

There are limited studies dealing with the applications of *G. mellonella* extracts as biological control elements, although the *G. mellonella* immune system is similar to the human system and has low cost.^46^ In this study, we have made the first attempt to test *G. mellonella* aqueous extracts in biomedical applications in combination with different minimal doses of gamma radiation. Certain enzymes have been reported to be secreted by *G. mellonella* larvae, such as acid phosphatase,^47^ protease,^48^ and lipase.^49^ Furthermore, it has been reported that gamma irradiation doses can regulate enzyme cascades in insects, leading to biological control,^50^ consistent with the present study, which shows that a 6 Gy irradiation dose leads to a beneficial secretion of enzymes that enhance the antibacterial and antitumour effects of samples.

## Conclusion

Further research is required to elucidate the exact mechanism of *G. mellonella* using different extraction systems to combat biological threats. This study suggests the potential use of a gamma-irradiated *G. mellonella* aqueous extract as an effective and safe biomedical agent, which promises to be an alternative tool to chemicals against known diseases, after further validation.

## Recommendations

*G. mellonella* has known applications; this study explored innovative medical uses of *G. mellonella* larvae as safe, alternative raw material that requires further verification.

## Source of funding

This research did not receive any specific grant from funding agencies in the public, commercial, or not-for-profit sectors.

## Conflict of interest

The authors have no conflict of interest to declare.

## Ethical approval

Not applicable.

## Authors contributions

MY and RS conceived and designed the study. RS, NAS, and BHA conducted the research, provided the research materials, and collected and organised the data. MY analysed and interpreted the data. MY wrote the initial and final drafts of the article. All the authors have critically reviewed and approved the final draft, and are responsible for the content and similarity index of the manuscript.
